# Vitamin D in Human Immunodeficiency Virus Infection: Influence on Immunity and Disease

**DOI:** 10.3389/fimmu.2018.00458

**Published:** 2018-03-12

**Authors:** María Ángeles Jiménez-Sousa, Isidoro Martínez, Luz María Medrano, Amanda Fernández-Rodríguez, Salvador Resino

**Affiliations:** ^1^Unidad de Infección Viral e Inmunidad, Centro Nacional de Microbiología, Instituto de Salud Carlos III, Majadahonda, Spain

**Keywords:** vitamin D deficiency, human immunodeficiency virus, inflammation, immune activation, adaptive immunity, innate immunity

## Abstract

People living with human immunodeficiency virus (HIV) infection typically have hypovitaminosis D, which is linked to a large number of pathologies, including immune disorders and infectious diseases. Vitamin D (VitD) is a key regulator of host defense against infections by activating genes and pathways that enhance innate and adaptive immunity. VitD mediates its biological effects by binding to the Vitamin D receptor (VDR), and activating and regulating multiple cellular pathways. Single nucleotide polymorphisms in genes from those pathways have been associated with protection from HIV-1 infection. High levels of VitD and VDR expression are also associated with natural resistance to HIV-1 infection. Conversely, VitD deficiency is linked to more inflammation and immune activation, low peripheral blood CD4+ T-cells, faster progression of HIV disease, and shorter survival time in HIV-infected patients. VitD supplementation and restoration to normal values in HIV-infected patients may improve immunologic recovery during combination antiretroviral therapy, reduce levels of inflammation and immune activation, and increase immunity against pathogens. Additionally, VitD may protect against the development of immune reconstitution inflammatory syndrome events, pulmonary tuberculosis, and mortality among HIV-infected patients. In summary, this review suggests that VitD deficiency may contribute to the pathogenesis of HIV infection. Also, VitD supplementation seems to reverse some alterations of the immune system, supporting the use of VitD supplementation as prophylaxis, especially in individuals with more severe VitD deficiency.

## Vitamin D (VitD) Background

### VitD Metabolism

**Figure 1:**
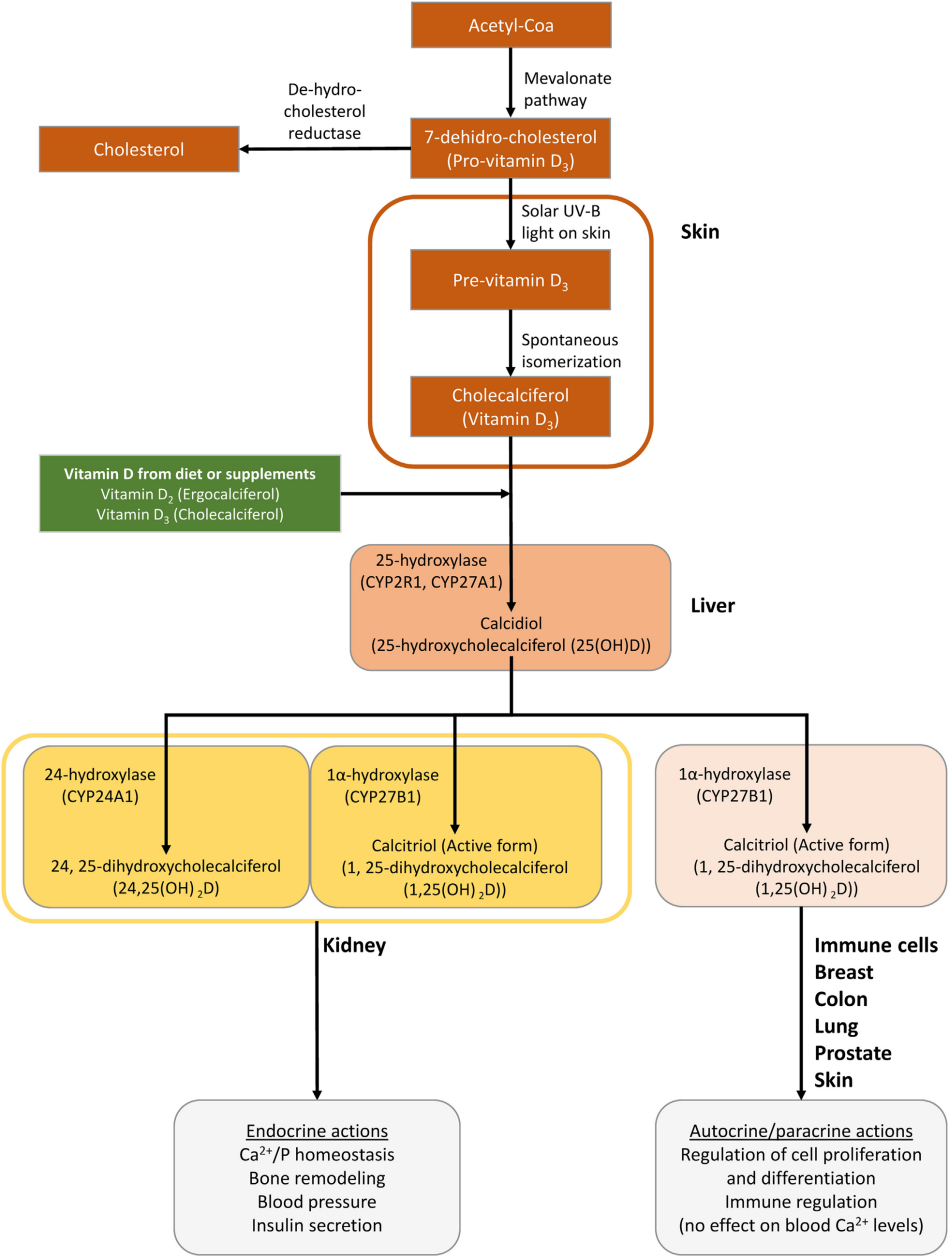
Schematic of the synthesis of vitamin D (VitD) in the body. Cutaneous 7-dihydrocholesterol is converted into preVitD_3_ following irradiation by ultraviolet light from the sun (2). Next, preVitD_3_ forms cholecalciferol (VitD_3_) by spontaneous isomerization. Subsequently, cholecalciferol is hydroxylated to 25-hydroxy-VitD (25(OH)D) or calcidiol, mainly in the liver, by the cytochrome P450 hydroxylase enzymes CYP27A1 and CYP2R1. Then, 25(OH)D is transported to the kidneys, where it is hydroxylated at the 1 alpha position by the 25-hydroxy-VitD-1 alpha hydroxylase (CYP27B1) to generate 1,25-dihydroxycholecalciferol [1,25 (OH)_2_D] or calcitriol, which is the metabolically active compound (1, 2). Hydroxyvitamin D-24-hydroxylase (CYP24A1) is the enzyme responsible for the multi-step catabolism of both 25(OH)D and 1,25 (OH)_2_D. The main product of 25(OH)D catabolism by CYP24A1 is 24,25-dihydroxycholecalciferol [24,25(OH)_2_D], which is less active than calcitriol and presumably represents a metabolite destined for excretion. Importantly, VitD is not only converted from 25(OH)D to 1,25 (OH)_2_D in the kidney but it is also activated locally by CYP27B1 in many tissues, including the brain, smooth muscle, breast, and prostate as well as cells of the immune system.

Vitamin D is a fat-soluble steroid synthesized from a cholesterol precursor (7-dehydrocholesterol), which has a chemical secosteroid structure (1). The major forms of VitD that are important to humans are VitD_2_ or ergocalciferol, synthesized from ergosterol in plants, and VitD_3_ or cholecalciferol synthesized naturally from cholesterol in animals (VitD_3_) (1, 2). They can be supplied to the body from the diet and VitD-fortified products, among other sources (1, 2). However, the main source of VitD for the human body is its synthesis in the skin. A flowchart describing VitD metabolism is represented in Figure 1.

### Transport and Mechanism of Action

**Figure 2:**
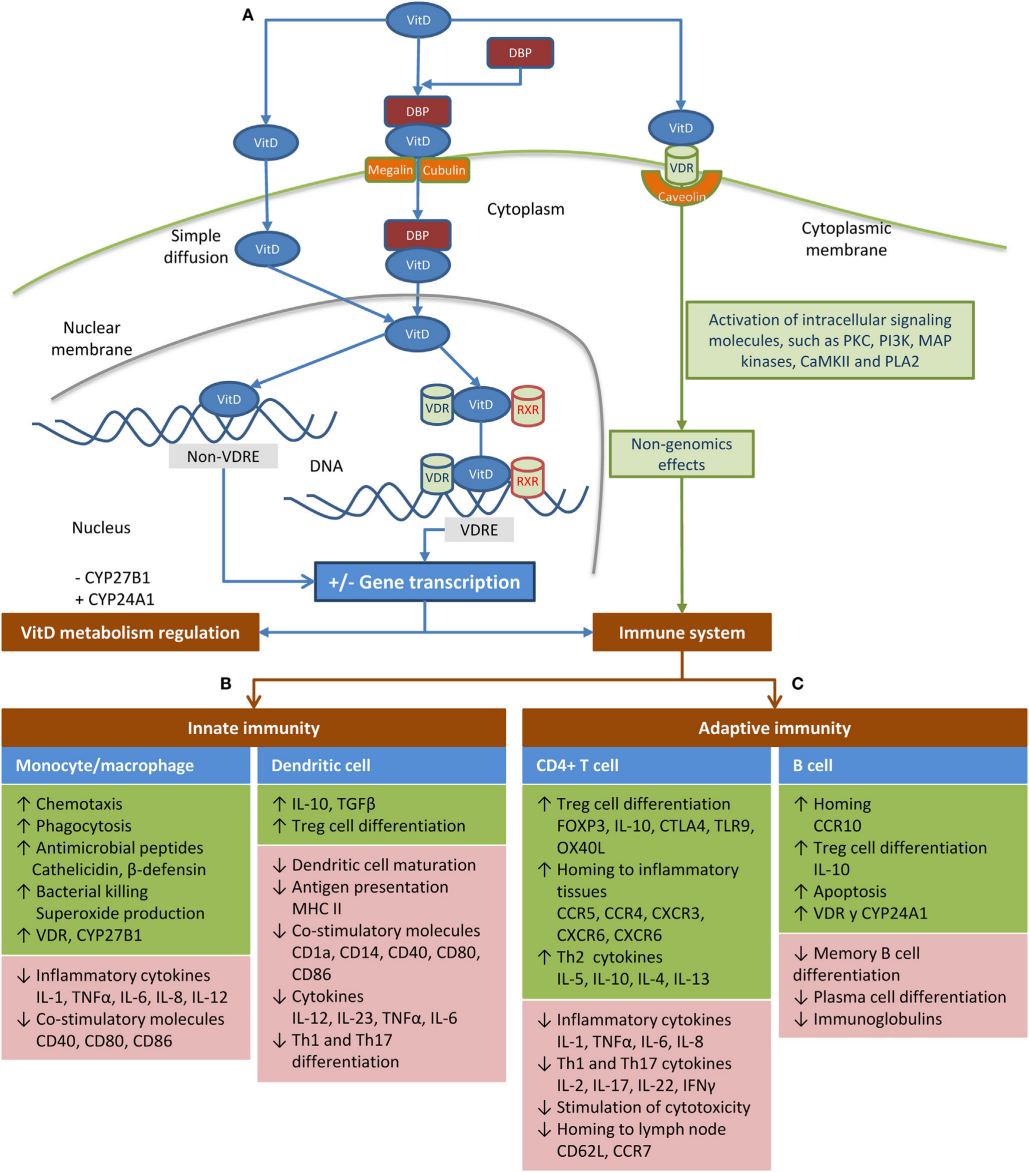
Schematic of transport and mechanism of action of vitamin D (VitD) in the body. **(A)** A small fraction of VitD circulates in the serum as a “free” steroid, having easy access to the intracellular compartment. The remaining VitD is transported in the blood while bound to the vitamin D-binding protein (DBP) (1, 2), which seems to critically regulate the bioavailability of VitD (7). This protein-bound fraction (bound to DBP) is actively transported into the cell by megalin or cubulin. Calcitriol is considered the main ligand of the vitamin D receptor (VDR) to trigger the effects of VitD, because its affinity is 1,000 times greater than calcidiol (8). When VitD binds to VDR in the nucleus of target cells, it forms a complex with the retinoic acid X receptor (RXR), which controls transcriptional activity of target genes. This heterodimer binds to VitD response elements (VDREs), a predefined promoter DNA sequence, initiating gene transcription processes, which covers around 5% of the human genome and 36 different cell types (4). However, there are genes regulated by VitD that do not contain VDREs (9). These genes may be regulated by microRNAs, phosphorylation, or other modifications of proteins, which affect their stability or the activity of proteases that target them (9). Additionally, non-genomic effects have been reported when the VDR is situated on the cell membrane (VDRm) complexed to caveolin (5), which immediately activates several intracellular pathways, such as mitogen-activated protein kinases, protein kinase C (PKC), protein kinase A, and Ca2+-calmodulin kinase II through the activation of several signaling molecules (5). VitD may reduce its synthesis by inhibiting CYP27B1 and increases its degradation by inducing CYP24A1 (6). **(B)** VitD modulates the function of monocytes/macrophages and dendritic cells (DCs) in response to infections. In monocytes/macrophages, 1,25(OH)_2_D leads to the expression of multi-target genes, among which are cathelicidin microbial peptide (10, 11), human β-defensin 4 (DEFB4) (12), and genes involved in autophagy and phagosome maturation, all of which are involved in the intracellular destruction of pathogens (7, 13). Furthermore, 1,25(OH)_2_D enhances the chemotactic and phagocytic capacity of macrophages (14). Moreover, VitD also promotes an anti-inflammatory response by inhibiting the maturation of DCs, resulting in a phenotype characterized by the downregulation of antigen presenting molecules (MHC-class II), costimulatory molecules (e.g., CD40, CD80, and CD86), and pro-inflammatory cytokines (e.g., IL-12 and IL-23); while an anti-inflammatory cytokine (IL-10) and T-cell inhibitory molecule (PD-1) are enhanced (15–22). Therefore, VitD induces hypo-responsiveness and allows a shift in the T-cell polarization from the pro-inflammatory Th1 and Th17 responses to a more tolerogenic Th2 response (16, 17, 20, 22–24), which leads to an altered alloreactive T cell activation (25). **(C)** VitD induces anti-inflammatory responses through direct effects on T-cells. Specifically, 1,25(OH)_2_D inhibits the proliferation of T-cells by blocking mitosis and IL-2 production (26, 27), limits the differentiation of Th1/Th17 cells, which favors Th2 differentiation (28–32), and induces the generation of IL-10 secretory Treg cells (32–34). Additionally, T-cell proliferation is significantly reduced when DCs are exposed to 1,25(OH)_2_D_3_ (16). T-cell cytokines also regulate VitD metabolism by monocytes. Thus, the Th1 cytokine IFN-γ induces CYP27B1, leading to the conversion of 25(OH)D to 1,25(OH)_2_D, whereas the Th2 cytokine IL-4 promotes upregulation of CYP24A1 (35). Stimulation of B-cells with 1,25(OH)_2_D leads to apoptosis, impaired plasma cell differentiation, decreased antibody production, inhibition of memory B-cell formation, and increased production of IL-10 (32, 36–41).

Transport and mechanism of action are shown in Figure 2A. A small fraction of VitD circulates in serum as “free” steroid and enters cells by simple diffusion. The remaining VitD in blood is transported bound to VitD-binding protein (DBP) (1, 2), which is able to bind the various types of VitD, albeit with different affinities. While DBP has a strong affinity for 25(OH)D, it has a weak affinity for 1,25(OH)_2_D. This low affinity together with the high affinity of the Vitamin D receptor (VDR) for 1,25(OH)_2_D makes 1,25(OH)_2_D the only ligand with direct access to the transcriptional signal transduction machinery (3). VitD binds to VDR in the nucleus, forming a complex with retinoic acid X receptor (RXR) and promotes gene transcription of several target genes by binding to VitD response elements (VDREs) (4). However, VitD can also regulate gene transcription *via* other mechanisms not related to VDREs. Additionally, VitD can enter the cell by binding to VDR situated on the cell membrane (VDRm), leading to non-genomics effects (5). The range of non-genomic effects is related to the cell-type and maturation status, but includes the modulation of growth factors and cytokines through cytosolic signaling pathways and effects on the activity of target transcription factors in the nucleus (5). Finally, VitD regulates its synthesis by a robust negative feedback mechanism (6).

The VitD system plays a global role in many physiopathological processes since VDR is expressed in tissues and cells nearly throughout the entire organism. The tissues with the highest VDR content are the intestine, kidney, parathyroid gland, and bone, all of which are associated with maintenance of calcium homeostasis (42). Immune cells also express VDR, and they are capable of metabolizing circulating 25-hydroxy-VitD (25(OH)D) to the active form 1,25-dihydroxycholecalciferol [1,25(OH)_2_D], indicating a regulatory role of VitD in both the innate and adaptive immune systems (43, 44). Additionally, the effect of VitD on the immune response by binding to VDR is also present in many other cells, such as keratinocytes, bronchial/gastrointestinal epithelial cells, decidua, and trophoblastic cells (45).

### VitD Levels: Measurement and Cut-Off Points

The quantification of 25(OH)D in serum or plasma is the fastest and most accurate way to measure VitD levels in the body. However, this method has some drawbacks due to the hydrophobic nature of VitD, its high affinity to DBP, and the low concentration in blood (46).

The measurement of 25(OH)D levels is performed mainly *via* two different methodologies (46, 47): (a) competitive immunoassays, such as competitive protein-binding assays or radioimmunoassays, which do not differentiate between 25(OH)D_2_ and 25(OH)D_3_ isoforms; and (b) tests based on high-performance liquid chromatography and direct detection with liquid chromatography tandem-mass spectrometry (LC-MS/MS), which are highly sensitive and allow for the independent quantification of 25(OH)D_2_ and 25(OH)D_3_.

Vitamin D levels are expressed in nanogram per milliliter (ng/mL) or nanomol/liter (nmol/L). VitD deficiency in adults is considered to be when total 25(OH)D levels are <25 nmol/L (10 ng/mL) and inadequate/insufficient if levels are <75 nmol/L (30 ng/mL); while >75 nmol/L (30 ng/mL) is considered to be a normal healthy level (47, 48). Suboptimal VitD levels have been reported in rickets, osteomalacia, and non-skeletal diseases. There is wide variability in the prevalence of VitD deficiency across different patient groups. Regarding human immunodeficiency virus (HIV), conflicting data have been found. Some authors have described that there is no evidence of higher VitD deficiency in HIV-infected patients compared to non-HIV adults (49). However, others have described that VitD deficiency was more prevalent in HIV-positive than in HIV-negative individuals (50).

There is a lack of standardization regarding reference materials and reference methods, which makes it difficult to compare results across different laboratories (47, 51). However, the accuracy of results as well as particular aspects of 25(OH)D and 1,25(OH)_2_D methods (linearity, specificity, and the effect of anticoagulants) are being assessed by the large group of experts comprising the VitD External Quality Assessment Scheme (DEQAS). DEQAS has a close link to the Vitamin D Standardization Program, which promotes the standardized laboratory measurement of 25(OH)D (52).

Vitamin D deficiency is a major public health problem around the world in all age groups, even in countries closer to the equator with adequate UV radiation and in industrialized countries where VitD is typically supplemented (53). The prevalence of VitD deficiency (<25 nmol/L) is between 5 and 15%, and hypovitaminosis D (<75 nmol/L) is from 50 to 75% in high-income countries (53). This deficiency is directly involved in bone pathologies (rickets, osteoporosis, and osteomalacia). Additionally, there is growing evidence supporting its association with many other “non-classical” disorders not related to the bones, such as cardiovascular disease, cancer, multiple sclerosis, metabolic disorders, and infectious diseases (54). In fact, VitD deficiency is related to an increased incidence and severity of *Mycobacterium tuberculosis* (TB), HIV, and hepatitis C virus (HCV) infection (55, 56).

## VitD in HIV Infection

### VitD Deficiency in HIV-Infected Patients

In a recent review article, Mansueto et al. showed that the prevalence of VitD deficiency ranges from 70 to 85% in HIV-infected patients, based on a large number of epidemiological articles that reported data of hypovitaminosis D with varying thresholds and a broad geolocalization of patients (56). VitD deficiency may be due to different reasons in these patients. On the one hand, there are many non-HIV-related risk factors for VitD deficiency, such as sex (females have higher risk), advanced age, limited sunlight exposure, skin pigmentation, black ethnicity, low levels of dietary VitD intake, gastrointestinal absorption disorders, liver and kidney diseases, higher body mass index, diabetes mellitus, and alcohol consumption (13, 56). These risk factors affect both HIV-positive and HIV-negative cohorts in a similar manner (57, 58). On the other hand, several HIV-related factors may lead to VitD deficiency. HIV infection itself leads to chronic inflammation and immune activation, and patients with VitD deficiency have been found to have increased IL6 and TNFα levels as well as activated monocyte phenotypes (59). Additionally, chronic inflammation may be responsible for impaired 1α-hydroxylase activity in the kidneys, resulting in reduced production of 1,25(OH)_2_D by blocking the PTH-stimulated conversion of 25(OH)D to 1,25(OH)_2_D (56, 60). Additionally, comorbidities, infectious complications, and hospitalizations of HIV-infected patients lead to reduced sun exposure, malnutrition, and diminished oral intake of VitD-rich foods (56, 61). In this regard, injection drug users infected with HIV suffer a disproportionate burden of VitD deficiency since they often have poor nutrition, limited and delayed access to health care, and a higher prevalence of comorbidities and infectious diseases (62). Finally, protease inhibitors (PIs) and non-nucleoside reverse transcriptase inhibitors (NNRTIs) seem to have an impact on VitD metabolic pathways. PIs seem to reduce 25(OH)D conversion to 1,25(OH)_2_D and NNRTIs seem to increase 25(OH)D catabolism, since low 25(OH)D levels have been seen in patients treated with these drugs (56).

### VitD Deficiency and Genetic Background

Several single nucleotide polymorphisms (SNPs) in *DBP* gene seem to influence plasma levels of VitD (63, 64). *DBP* gene variants associated with reduced 25(OH)D levels were also associated with reduced DBP levels. In this setting, it has been hypothesized that altered DBP levels could affect the delivery of 1,25(OH)2D to target tissues, as well as the removal of VitD metabolites from circulation (63). A significant association has been found between rs222020 and rs2282679, which are in low linkage disequilibrium (LD), and the variation of serum 25(OH)D levels in healthy populations (64). Besides, rs222020 G allele, which has been associated with VitD deficiency, has been related to unfavorable outcome in HIV infection (65). Moreover, many SNPs located in genes related to the VitD pathway (DHCR7, CYP2R1, CYP3A4, CYP27A1, DBP, LRP2, CUB, CYP27B1, CYP24A1, VDR, and RXRA) are linked to a large number of non-skeletal health problems, especially infectious and autoimmune-related diseases (66). For example, the VDR rs1544410 G allele is related to the delayed progression of acquired immunedeficiency syndrome (AIDS) and increased resistance to HIV infection, which appears to be related to an increased response to VitD (67, 68). Additionally, *VDR* haplotypes conformed by rs11568820, rs4516035, rs10735810, rs1544410, and rs17878969 polymorphisms are also associated with protection against HIV infection. In this context, the protective haplotype has been related to a lower efficiency of VitD signaling, suggesting that an altered VitD pathway confers protection against HIV transmission. The exact mechanism of these polymorphisms is unclear. However, it is thought that *VDR* rs1544410 could reduce VDR messenger RNA production and stability, rs10735810 T allele might lead to a low transactivation capacity of the VDR protein, preventing normal VDR function, and *VDR* promoter rs4516035 could be biologically crucial to the immune system (69). Additionally, it is important to take into account that unknown functional genetic variants in LD with the known ones could be responsible for the regulation of serum 25(OH)D levels and the disease outcome.

## VitD and the Immune System

### VitD, Innate Immunity, and HIV Infection

Vitamin D is involved in host defense *via* an autocrine pathway in human monocytes and macrophages following stimulation of toll-like receptors (TLRs)2/1, TLR4, the receptor of interferon gamma (IFN-γ) or CD40 (70, 71). These receptors initiate a signaling cascade that induces upregulation of the VDR and CYP27B1, which leads to the conversion of 25(OH)D to 1,25(OH)_2_D. Binding of 1,25(OH)_2_D to the VDR leads to the expression of multitarget genes, which modulate the function of monocytes/macrophages during infection (see Figure 2B). Moreover, VitD prevents the excessive inflammatory response to infectious diseases by inhibiting the maturation of dendritic cells (DCs) (7, 13). Note that DCs also express VDR, as well as CYP27A1 and CYP27B1, thereby generating locally bioactive 1,25(OH)_2_D (72, 73). However, Kundu et al. described that human monocyte-derived DCs convert 25(OH)D to 1,25(OH)_2_D significantly less than macrophages, likely due to the fact that DCs mostly express a truncated CYP27B1 transcript, which may lead to a deficiency in VitD activation (74).

High levels of VitD and its receptor seem to be associated with a natural resistance to HIV-1 infection. This may stem from the upregulation of anti-inflammatory IL-10 and induction of anti-HIV-1 defensins in mucosa of HIV-1-exposed seronegative individuals (75). In addition, the expression of VDR is positively correlated with the expression of several anti-HIV molecules [such as elafin, TRIM5, cathelicidin microbial peptide (CAMP), HAD-4, and RNase7], which are linked to natural resistance to HIV-1 infection (76). Furthermore, it has been described that exogenous 1,25(OH)_2_D in monocytes decreases susceptibility to HIV infection by inhibiting viral entry, reducing surface CD4 expression and limiting monocyte proliferation (77, 78). Also, it has been demonstrated that TLR8 agonists inhibit HIV infection through a VitD- and CAMP-dependent autophagic mechanism in human macrophages (79). Furthermore, it has also been shown that VitD triggering autophagy in macrophages significantly inhibits replication of HIV-1 in a dose-dependent manner (80). However, there are other studies with contradictory data, in which VitD was found to increase HIV replication in monocytes both from patients and cell line clones (81–83).

In contrast, VitD deficiency is associated with greater inflammation (upregulation of CXCL10, IL-6, TNF-α, and D-dimer) and activated monocyte phenotypes (CX3CR1+ and CCR2+) in HIV-infected patients (55, 59, 84), which have been related to tissue dysfunction, comorbidity development, AIDS progression, and death in HIV-infected people (85, 86). In addition, chronic inflammation may also induce hypovitaminosis D (7). Thus, inflammatory processes involved in the appearance and clinical course of disease may reduce 25(OH)D levels, which would explain the low VitD status in a wide range of disorders in the general population (54) and in HIV-infected patients (7, 56). Legeai et al. found that severe VitD deficiency is associated with low CD4 counts and increased markers of inflammation in combination antiretroviral therapy (cART)-naïve HIV-infected patients (87). However, it is important to note that high 25(OH)D levels have also been recently associated with unexpectedly high levels of proinflammatory cytokines in HIV patients on cART therapy (88). Additionally, LPS and HIV gp120 upregulate the expression of CYP27B1 and CYP24A1 in monocytes and macrophages, leading to hypovitaminosis D in HIV-infected individuals and a reduction in mRNA expression of VDR and the antiviral peptides PI3 and CAMP (89, 90).

The restoration of VitD levels to normal values may minimize both ongoing inflammation and the complications of HIV and cART associated with chronic inflammation (13). For example, VitD supplementation stimulates expression of CAMP and improves antibacterial immunity in monocyte cultures and plasma from HIV-infected subjects (91, 92). VitD supplementation decreased markers of monocyte activation in HIV-infected patients (93). *In vitro*, VitD exposure improves the chemotactic activity of macrophages in AIDS patients (94). In addition, HIV-infected patients suffer from bacterial translocation, which is an effect of intestinal barrier damage caused by HIV itself (95). Recent studies have demonstrated the role of VitD in regulating host–bacteria interactions, intestinal innate immunity, and homeostasis (96). However, the rationale for VitD supplementation to reduce microbial translocation and systemic inflammation in the HIV population is controversial. For instance, no association between VitD levels and markers of microbial translocation was found in HIV-infected patients (97), while optimal VitD plasma levels have been associated with lower bacterial DNA translocation in HIV/HCV-coinfected patients (98). These differences between studies could be due to a high percentage of HIV/HCV-coinfected patients with advanced fibrosis/cirrhosis in the latter study, since severity of liver disease has been related to VitD deficiency (99) and bacterial translocation (100) in HIV/HCV-coinfected patients. The lack of consensus guidelines about VitD supplementation in HIV infection underlies the need for robust studies to critically evaluate the potential benefits of VitD supplementation in these patients.

Additionally, VitD has also been related to other infectious diseases in HIV-infected patients. VitD rescues impaired TB-mediated TNF release in macrophages of HIV-infected patients through an enhanced TLR signaling pathway (101). Additionally, high VitD levels have been associated with protection against the development of immune reconstitution inflammatory syndrome (IRIS) events (102) and decreased incidence of pulmonary tuberculosis and mortality among HIV-infected patients (103). However, a recent meta-analysis shows that there are other studies, particularly in HIV/TB-coinfected African patients receiving cART, which have not found any association between lower VitD levels and IRIS. Besides, they showed that VitD deficiency was not associated with an increased risk of TB in African HIV-infected patients (104). In regards to other infectious diseases, VitD levels have not been associated with better immune response to hepatitis B or pneumococcal vaccination in HIV-infected patients (105).

### VitD, Adaptive Immunity, and HIV Infection

Vitamin D may indirectly affect T-cell responses *via* modulation of the DC phenotype and its stimulatory capacity toward T cells (106). Additionally, both naïve and resting memory T-cells express VDR at low levels, which suggests that VitD also acts directly on these T-cells (28, 107). T-cell activation increases the expression of VDR and CYP27B1, which allows 25(OH)D to be converted into 1,25(OH)_2_D to modulate effector functions of VitD (108). VitD suppresses the Th1, Th17, and Th2 profile of cytokine production, thereby altering T cell phenotype and function (106) (see Figure 2C). The effect of VitD on B-cells is thought to be the modulation of Th cells (106). However, human B-cells also express VDR and CYP27B1, which are upregulated upon activation, suggesting that B-cells may also be susceptible to 1,25(OH)_2_D stimulation. VitD induces hypo-responsiveness of B-cells and interferes with human plasma cell differentiation (106) (see Figure 2C).

Vitamin D induces antiviral gene expression, reduces the viral co-receptor CCR5 on CD4+ T-cells, and promotes an HIV-1-restrictive CD38+HLA-DR+ immunophenotype in *in vitro* assays, leading to HIV-1 infection inhibition in T cells (109). Likewise, VitD reduces the ability of TNFα to upregulate the transcription of HIV RNA from latently infected CD4+ cells (110). Thus, low levels of VitD are related to high HIV viral load in plasma (111–113), decreased CD4+ T-cells in peripheral blood (114, 115), rapid AIDS progression, and lower survival in HIV-infected patients (61, 84, 116–118).

Vitamin D deficiency seems to have no influence on T-cell and B-cell subset distribution in HIV-infected patients, but VitD supplementation is related to reduced immune activation levels (CD8+CD38+ and CD8+Ki67+) (119), and increased frequencies of antigen-specific T-cells expressing macrophage inflammatory protein-1β, an important anti-HIV blocking chemokine (91). Besides, Eckard et al. reported that VitD supplementation reduces immune activation and T-cell exhaustion, serving as an adjuvant therapy to cART in HIV-infected patients (93). However, high levels of 1,25(OH)_2_D have been associated with an expansion of activated CD4+ cells and Tregs in HIV-infected patients (120). In addition, high levels of 1,25(OH)_2_D increase the percentage of CD4+ T-cells expressing TIM-3 protein (a Th1-cell inhibitor and anti-HIV-1 molecule) in *in vitro* assays with peripheral blood mononuclear cells of HESN (89).

Vitamin D deficiency is also related to impaired CD4+ T-cell count recovery in HIV-positive patients on cART (121–123). Moreover, VitD levels are positively associated with CD4+ T-cell recovery after 24 weeks of VitD supplementation (115), suggesting a potential benefit of VitD supplementation on immunologic recovery during cART. However, there are other reports that showed no effect of VitD supplementation on CD4+ T-cells in HIV-infected adults (116, 124) or children (125).

## Conclusion

Vitamin D deficiency may contribute to the pathogenesis of HIV infection by negatively modulating the innate and adaptive immune responses. Low VitD levels promote inflammation and activation of the immune system, which could increase the risk of non-AIDS-related comorbidity and mortality in the HIV-infected population. Moreover, exogenous VitD supplementation could be used as prophylaxis, as it appears to reverse some alterations of the immune system due to VitD deficiency, especially in individuals with more severe deficiency. Finally, there is a paucity of studies exploring the association between VitD levels and HIV infection.

## Author Contributions

Conceptualization, visualization, and supervision: SR. Resources and data curation: MJ-S, AF-R, LM, and IM. Writing—original draft preparation: MJ-S, IM, and SR. Writing—review and editing: AF-R and LM.

## Conflict of Interest Statement

The authors declare that the research was conducted without any commercial or financial relationships that could be construed as a potential conflict of interest.
